# Longitudinal [^18^F]FDG-PET/CT analysis of the glucose metabolism in *ApoE*-deficient mice

**DOI:** 10.1186/s13550-020-00711-4

**Published:** 2020-10-07

**Authors:** Angela Kuhla, Lou Meuth, Jan Stenzel, Tobias Lindner, Chris Lappe, Jens Kurth, Bernd J. Krause, Stefan Teipel, Änne Glass, Guenther Kundt, Brigitte Vollmar

**Affiliations:** 1 Institute for Experimental Surgery, Rostock University Medical Center, Schillingallee 69a, 18057 Rostock, Germany; 2 Core Facility Multimodal Small Animal Imaging, Rostock University Medical Center, Rostock, Germany; 3 Institute of Diagnostic and Interventional Radiology, Pediatric and Neuroradiology, Rostock University Medical Center, Rostock, Germany; 4 German Center for Neurodegenerative Diseases (DZNE), Rostock, Greifswald, Germany; 5 Department of Nuclear Medicine, Rostock University Medical Center, Rostock, Germany; 6 Department of Psychosomatic Medicine, Rostock University Medical Center, Rostock, Germany; 7 Institute for Biostatistics and Informatics in Medicine and Ageing Research, Rostock University Medical Center, Rostock, Germany

**Keywords:** Cerebral glucose metabolism, [^18^F]FDG-PET/CT imaging, Proton magnetic resonance spectroscopy, Brain stem, *ApoE-*deficiency

## Abstract

**Background:**

Strong line of evidence suggests that the increased risk to develop AD may at least be partly mediated by cholesterol metabolism. A key regulator of cholesterol transport is the Apolipoprotein E4 (ApoE4), which plays a fundamental role in neuronal maintenance and repair. Impaired function of ApoE4 may contribute to altered cerebral metabolism leading to higher susceptibility to neurodegeneration.

**Methods:**

To determine a possible link between ApoE function and alterations in AD in the brain of Apolipoprotein E-deficient mice (*ApoE*−/−) in a longitudinal manner metabolic and neurochemical parameters were analyzed. Cortical metabolism was measured by 2-deoxy-2-[^18^F]fluoroglucose ([^18^F]FDG)-PET/CT and proton magnetic resonance spectroscopy (^1^H-MRS) served to record neurochemical status.

**Results:**

By using [^18^F]FDG-PET/CT, we showed that brain metabolism declined significantly stronger with age in *ApoE*−/− versus wild type (wt) mice. This difference was particularly evident at the age of 41 weeks in almost each analyzed brain region. In contrast, the ^1^H-MRS-measured *N*-acetylaspartate to creatine ratio, a marker of neuronal viability, did not decline with age and did not differ between *ApoE*−/− and wt mice.

**Conclusion:**

In summary, this longitudinal in vivo study shows for the first time that *ApoE*−/− mice depict cerebral hypometabolism without neurochemical alterations.

## Background

The Apolipoprotein E4 (ApoE4) allele is the strongest single genetic risk factor for sporadic Alzheimer’s disease (AD) [1, 2]. The mechanism of the ApoE4-associated disposition to AD is still not fully understood. A strong line of evidence suggests that the increased risk to develop AD may at least be partly mediated by cholesterol metabolism [3]. ApoE is the most prevalent brain apolipoprotein and plays a fundamental role in neuronal maintenance and repair [4], including cholesterol-derived synaptogenesis [5, 6]. Impaired function of ApoE4 leads to disordered cholesterol homeostasis contributing to increased susceptibility to neuroinflammation [7] and in consequence to neurodegeneration [8]. Accordingly, ApoE-deficient mice (*ApoE*−/−), which are characterized by hypercholesterolemia [9] and hypertriglyceremia (own unpublished data), showed a significant impairment of cognitive function [10] potentially related to AD pathology [11–13] such as tauopathy [11, 13].

In human studies, the ApoE4 genotype is associated with reduced cortical metabolism in AD predilection sites, such as the posterior cingulate gyrus. Cortical hypometabolism, which is not only observed in clinically manifest stages of AD, but already in pre-clinical stages can be measured by 2-deoxy-2-[^18^F]fluoroglucose ([^18^F]FDG)-PET/CT [14, 15]. Furthermore, Reiman et al. [16] found that the human APOEɛ4 gene dose correlated with [^18^F]FDG-PET/CT measurements of hypometabolism in AD-affected brain regions in a cognitively normal cohort, and postulated to use PET/CT as a pre-symptomatic endophenotype to help assess putative modifiers of AD risk. Moreover, [^18^F]FDG-PET/CT is a widely used tool in pre-clinical studies investigating AD pathology [17]. Here, a pre-clinical study reported that mice carrying the human APOE 4 isoform (hApoE4-TR) showed decreased [^18^F]FDG uptake [18]. Complementary to [^18^F]FDG-PET/CT, proton magnetic resonance spectroscopy (^1^H-MRS) allows characterization of neurochemical alterations in AD brains [19]. *N*-Acetylaspartate (NAA) is considered to reflect neuronal mitochondrial function [20, 21]. Decreased levels of NAA may reflect alterations of neuronal functional viability. Since alterations in NAA can be detected before the clinical appearance of dementia [22, 23], reduced NAA level may potentially serve as an early biomarker [24, 25].

In the current study we used small animal [^18^F]FDG-PET/CT and NAA levels from ^1^H-MRS to identify the effects of ApoE deficiency on cortical metabolism and neurochemical changes in a transgenic mouse model. We hypothesized that ApoE deficiency may lead to alterations of metabolism and neuronal function resembling effects in transgenic AD models as well as in human AD studies. In addition, we expected that the outcome of this study would help us to establish these imaging markers as potential read outs to predict in future the effects of interventions into ApoE-related mechanisms of risk propagation, such as cholesterol metabolism or neuroinflammation.

## Methods

### Animals

**Fig. 1:**
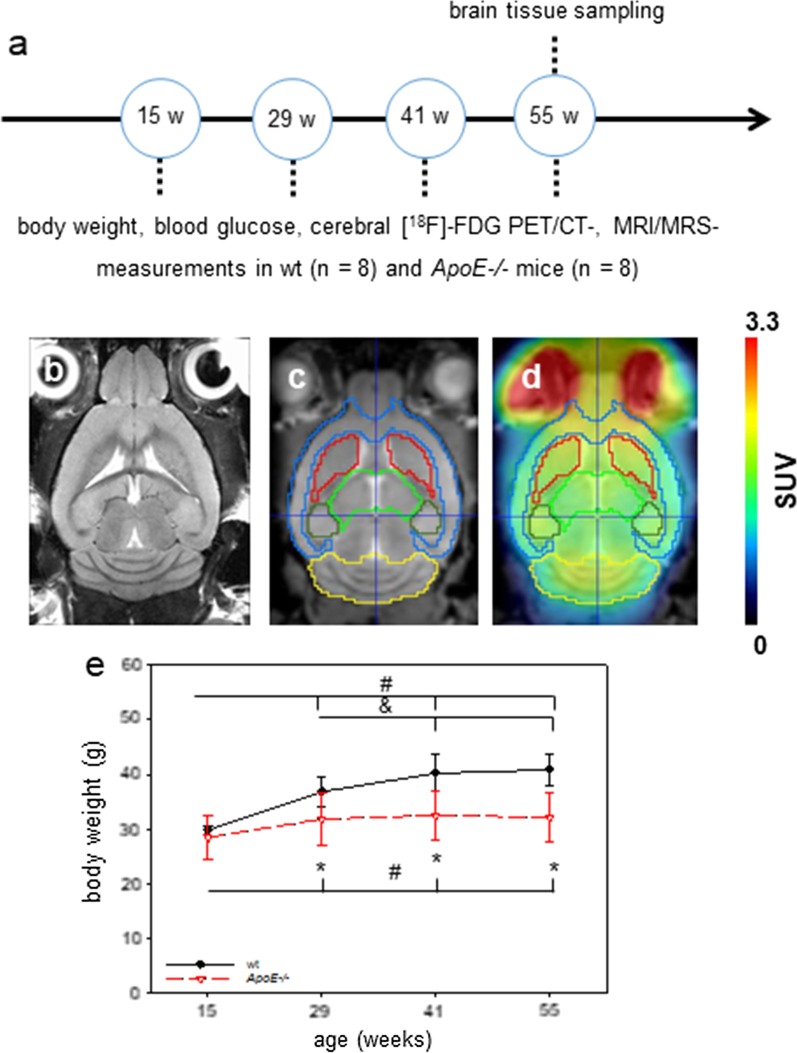
**a** Schematic illustration of the experimental design. Body weight, blood glucose levels as well as cerebral [^18^F]FDG-PET/CT and MRI/MRS measurement were evaluated at the age of 15, 29, 41, and 55 weeks (w) in wild type (wt, *n* = 8) and Apolipoprotein E-deficient (*ApoE*−/−, *n* = 8) mice. **b**, **c** Transversal T2 weighted TurboRARE (in-plane resolution: 65 × 65 µm, slice thickness 500 µm) and transversal T1 weighted isotropic 3D Flash (resolution: 120 × 120 × 120 µm) with M. Mirrione based mouse VOI template overlay (cortex—blue, striatum—red, thalamus—light green, hippocampus—dark green, cerebellum—yellow, brain stem—brown) of a wild type mouse. **d** Transversal [^18^F]FDG-PET/CT and MRI with M. Mirrione VOI template fusion. **e** Body weights were measured directly before [^18^F]FDG-PET/CT and MRI/MRS measurements. Values are given as mean ± SD; ANOVA for repeated measurements followed by Holm-Sidak comparison test: *p < 0.05 versus wt, #*p* < 0.05 versus 15 weeks, and *p* < 0.05 versus 29 weeks

Male C57BL/6 (ApoE competent; wild type mice, wt, *n* = 8) and male ApoE-deficient mice (*ApoE−/−*, *n* = 8) with identical genetic background (Charles River Wiga, Sulzfeld, Germany) were studied longitudinally at the ages of 15, 29, 41 and 55 weeks. Body weight and blood glucose level were measured before each MRI/MRS and [^18^F]FDG-PET/CT measurement (Fig. 1a) at the indicated time points. Mice were housed in groups in standard cages with enrichments in a temperature-controlled room (22 °C ± 2 °C) on a 12 h light/dark cycle (light turned on at 06:00 a.m.) with free access to food (4.2% fat) and water under specified pathogen free conditions. All procedures were conducted in accordance with animal protocols approved by the local Animal Research Committee (Landesamt für Landwirtschaft, Lebensmittelsicherheit und Fischerei (LALLF) of the state Mecklenburg-Western Pomerania (LALLF M-V/TSD/7221.3-1.1-009/15). All animals received care according to the German legislation on protection of animals and the Guide for the Care and Use of Laboratory Animals (European Directive 2010/63/EU). At the end of the experiment, all mice at the age of 55 weeks were sacrified by overdose of anesthesia, followed by harvest of brain tissues for immunhistological analysis.

### MR imaging

All mice were anesthetized by 1–3% isoflurane in 100% O2. The heads of the mice were placed with the animal's incisors secured over a bite bar and ophthalmic ointment was applied to the eyes. Animals were imaged in vivo with a T2-weighted Turbo-RARE (Rapid Acquisition with Relaxation Enhancement) and an isotropic T1-weighted FLASH sequence in a 7 T small animal MRI-scanner (BioSpec 70/30, gradient insert: BGA-12S, maximum gradient strength: 440 mT/m, Software interface: Paravision 6.01., Bruker BioSpin GmbH, Ettlingen, Germany) which was equipped with a 1H cryogenic, two elements, transmit/receive coil array. Animal welfare was ensured by employing a water driven warming mat as well as constant respiration and core body temperature monitoring.

### PET/CT imaging

Small animal PET/CT imaging scans were performed according to a standard protocol. Briefly, mice were not fasted and anesthetized by isoflurane (1–3%) supplemented with oxygen and received a mean dose of 17.36 ± 0.33 MBq [^18^F]FDG intravenously via a microcatheter placed in a tail vein. The protocol was adapted to Wong et al. [26] reporting no significant differences in the [^18^F]FDG uptake in mice between fasted and non-fasted state [26]. Moreover, the present study was designed according to own previous studies [13, 27] showing the neuroprotective effect of caloric restriction versus ad-libitum feeding in *ApoE*−/− mice. Similarly, interval fasting also caused neuroprotective effects. Therefore, fasting could be viewed as an intervention that affects brain metabolism. To avoid this effect and to maintain the comparability to previous studies the mice were not fasted. Likewise the scanning time was derived from Wong et al. [26] showing the highest plasma activity of [^18^F]FDG in the first 30 min. Therefore, after an uptake time of 30 min, static PET scans in head-prone position were acquired for 30 min using a small animal micro PET/CT scanner (Inveon PET/CT Siemens, Knoxville, TN, USA). Throughout the imaging session, respiration of the mice was controlled and body temperature was constantly kept at 38 °C via heating pad. The PET image reconstruction method consisted of a 2-dimensional ordered subset expectation maximization algorithm (2D-OSEM) with four iterations and 6 subsets, although 3D-OSEM is more suitable for mouse brain imaging analysis. However, the protocol was used in accordance with Poisnel et al. [28] studying [^18^F]FDG uptake in the APP/PS1 mice. Attenuation correction was performed on the basis of whole body CT scan and a decay correction for fluorine-18 was applied. PET/CT images were also corrected for random coincidences, dead time and scatter.

### PET/CT-data analysis

Image processing was performed using PMOD software (version 3.7; PMOD Technologies LLC, Zürich, Switzerland). The brain PET images of each mouse were spatially co-registered to a mouse MRI brain template (Fig. 1b, T2 weighted Mouse M. Mirrione template) which is included in the PMOD software. The individual PET images were first co-registered with their individual CT and the head areas were cropped. To compensate differences in positioning between CT and MRI measurements, the animal specific CT images of the cropped brain regions were rigidly transformed to match corresponding MRI T1 brain images. Afterwards, the MRI images of each mouse were transformed to the Mouse M. Mirrione template by transformations (Fig. 1c). Finally, the PET/CT transformation was normalized to the CT/MRT T1 and the MRI T1/Mouse M. Mirrione template transformation. The processed PET images were subsequently co-registered with the mouse brain volume-of-interest (VOI) template (Mouse Mirrione atlas, Fig. 1d), included in the PMOD software, and tracer uptake values were extracted for each delineated VOI. For each VOI standardized uptake values (SUVs) were acquired from cortex, hippocampus, striatum, thalamus, and brain stem. Those SUVs were normalized to SUVs of the brain stem and given as SUVRs.

### MR spectroscopy

**Fig. 2:**
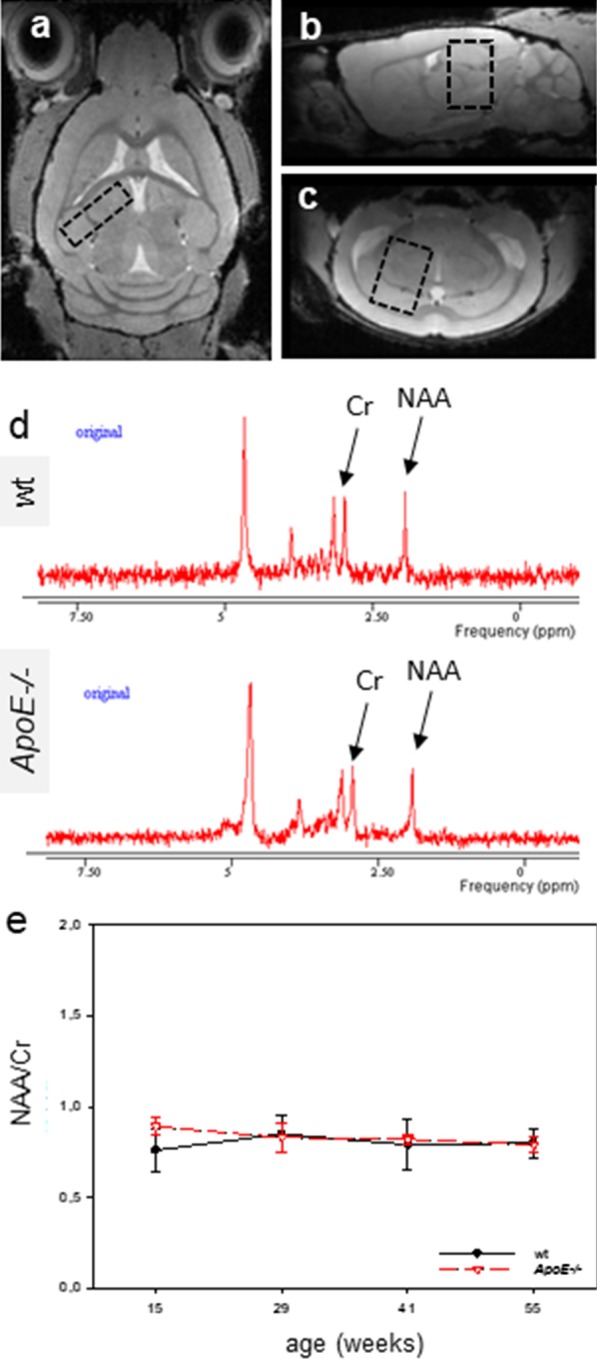
Transversal (**a**), sagittal (**b**) and coronal (**c**) T1 weighted MRI images including the position of the spectroscopy voxel (black dashed box) of a wild type (wt) mouse. An example of a MRS spectrum of a wt (upper) and an Apolipoprotein E-deficient (*ApoE*−/−) mouse (lower) as derived from the voxel of interest is shown in (**d**). Two prominent metabolites, e.g. *N*-Acetylaspartate (NAA resonates at 2.0 ppm) and creatine (Cr at 3.0 ppm) are evident and were further evaluated. **e** Diagram of the NAA/Cr ratio in wt and *ApoE*−/− mice. Values are given as mean ± SD

The imaging protocol included a morphological, respiration triggered, transversal T2-weighted (T2w) RARE (Rapid Acquisition with Relaxation Enhancement) sequence with following parameters: TE/TR: 39/2200 ms; FoV: approx. 13 mm × 17 mm; matrix: 200 pix × 260 pix; voxel size: 0.065 mm × 0.065 mm × 0.5 mm, approx. 18 slices. In addition, T2w images with similar resolution in the transversal (a), sagittal (b) and coronal (c) plane were acquired for ^1^H-MRS voxel placement. Additionally, a T1w FLASH sequence (Fig. 2a–c) was scanned for PET/CT data co-registration with following parameters: TE/TR: 8/80 ms; flip angle: 10°, FoV: 17.12 mm × 14.2 mm × 8.4 mm; matrix: 143 pix × 117 pix × 70 pix; voxel size: 0.12 mm × 0.12 mm × 0.12 mm, Avg.: 2. Respiration triggered ^1^H-MRS was carried out by means of the Stimulated Echo Acquisition Method (STEAM) with outer volume suppression and a voxel volume of approximately 10 mm^3^ (placed in the cortex and hippocampus, see Fig. 2a–c). The following parameters were used: acquisition bandwidth: 4.9 kHz; TE/TR: 135/1500 ms; mixing time 11,75 ms, 512 averages; acquisition time: 13 min. Each free induction decay was recorded with 2048 complex points. The water signal was suppressed using the variable pulse power and optimized relaxation delays scheme (VAPOR) [29]. Based on B_0_-field map measurements, the linewidth/spectral resolution was optimized by adjustments of first- and (if necessary) second-order shims, resulting in an average full width half maximum linewidth of the unsuppressed water peak between 10 and 25 Hz. MRS spectrum was derived from voxel of interest and was visualized via jMRUI. As previously described by Kuhla et al. [30], spectra were analyzed with the spectroscopy package. Metabolite ratios were calculated based on the area under the corresponding fitted curves for *N*-Acetylaspartate (NAA 2.0 ppm) and creatine (Cr 3.0 ppm). For quantitation of the metabolites the Hankel-Lanczos Singular Value Decomposition (HLSVD) method with five components was applied [31].

### Immunhistochemistry

For the assessment of a tauopathy and of neuroinflammation in form of astrogliosis, AT8 and GFAP immunohistochemistry were performed. Brain tissue was fixed in 4% phosphate-buffered formalin and embedded in paraffin. From the paraffin-embedded tissue blocks, 4 µm thin sections were put on X-tra Adhesive Precleaned Micro Slides (Leica) and exposed to the antibodies: a mouse monoclonal anti-AT8 antibody (1:1.000, Invitrogen) and rabbit polyclonal anti-GFAP antibody (1:100; Abcam). For the development of the primary antibodies with DAB chromogen Universal LSAB® kits (System-HRP; DakoCytomation, Dako) were used according to the manufacturer’s instructions. The sections were counterstained with hemalaun and analyzed with a light microscope (Zeiss Axiovision, Jena). Images were acquired with a Color View II FW camera (Color View). Within the cortex (*n* = 8 of each mouse strain, *n* = 20 of visual fields), the number of anti-GFAP positive cells were manually counted and given as number per high power field (HPF).

### Statistical analysis

The statistics computed included mean, standard deviations, and standard error of mean for continuous variables and are presented as mean ± SD. Because measurements of SUVs or SUVRs were made several times (mice at 15, 29, 41, and 55 weeks of age) on the same sample within two independent genotype groups (wild type and *ApoE*−/− mice), we applied the GLM repeated measures analysis of variance (ANOVA) followed by post hoc comparison test (Holm-Sidak method). We tested main effects for the between-subject factor “genotype”, within-subject factor “time”, and the interaction of “genotype*time”. For the pairwise comparison statistical differences were determined using unpaired student-t test and in case of failed normality Mann–Whitney Rank Sum test was used. All *p*-values were derived from two-sided statistical tests and values of *p* < 0.05 were considered to be statistically significant. Statistical analysis was performed using the SigmaStat software package (Jandel Corporation, San Rafael, CA, USA). The results were presented with the program SigmaPlot 13.0 (Jandel Corporation, San Rafael, CA, USA).

## Results

### Body weight analysis

The body weight of both mouse strains increased significantly over the observation period of 55 weeks (within-subject type “time-effect”: *p* < 0.001; Fig. 1e). However weight gain with aging was significantly more pronounced in the wt mice compared to the *ApoE*−/− mice (interaction “genotype*time”: *p* < 0.001; Fig. 1e). Furthermore, the body weight of the *ApoE*−/− mice was in general lower (between subject factor "genotype”, *p* = 0.005) and was significantly reduced compared to the wt mice (for detailed statistics see legend of Fig. 1). Blood glucose measurements revealed no differences between both mouse strains at each examined time point whereas the values slightly decreased over time (Additional file 1: Fig. 1S, within-subject type “time-effect”; *p* = 0.032).

### Spectroscopy

Figure 2 shows a transversal (a), sagittal (b) and coronal (c) T2 weighted MRI image of a wt mouse including the position of the spectroscopy voxel (black dashed box). An example of a MRS spectrum as derived from the voxel of interest is shown in Fig. 2d. The NAA/Cr ratio did not differ between age or mouse type (Fig. 2e).

### [^18^F]FDG-PET/CT uptake values

**Fig. 3:**
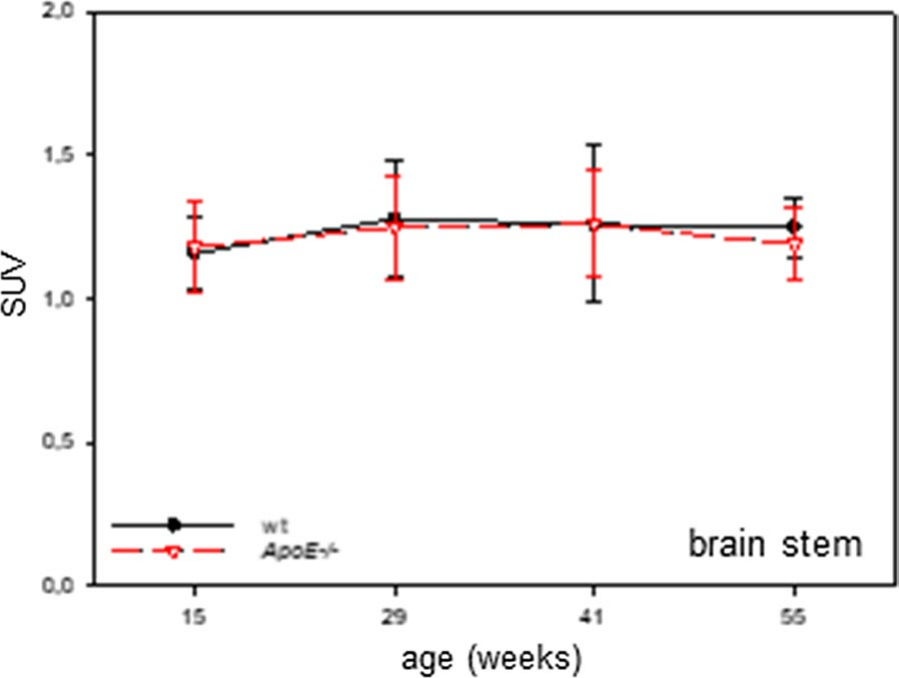
Quantification of [^18^F]FDG uptake in the brain stem given as absolute SUVs of wild type (wt; *n =* 8) and Apolipoprotein E-deficient (*ApoE*−/−; *n* = 8) mice at the age of 15, 29, 41, and 55 weeks. Notably, the brain stem showed neither age-dependent nor strain-dependent differences in [^18^F]FDG uptake. Values are given as mean ± SD

**Fig. 4:**
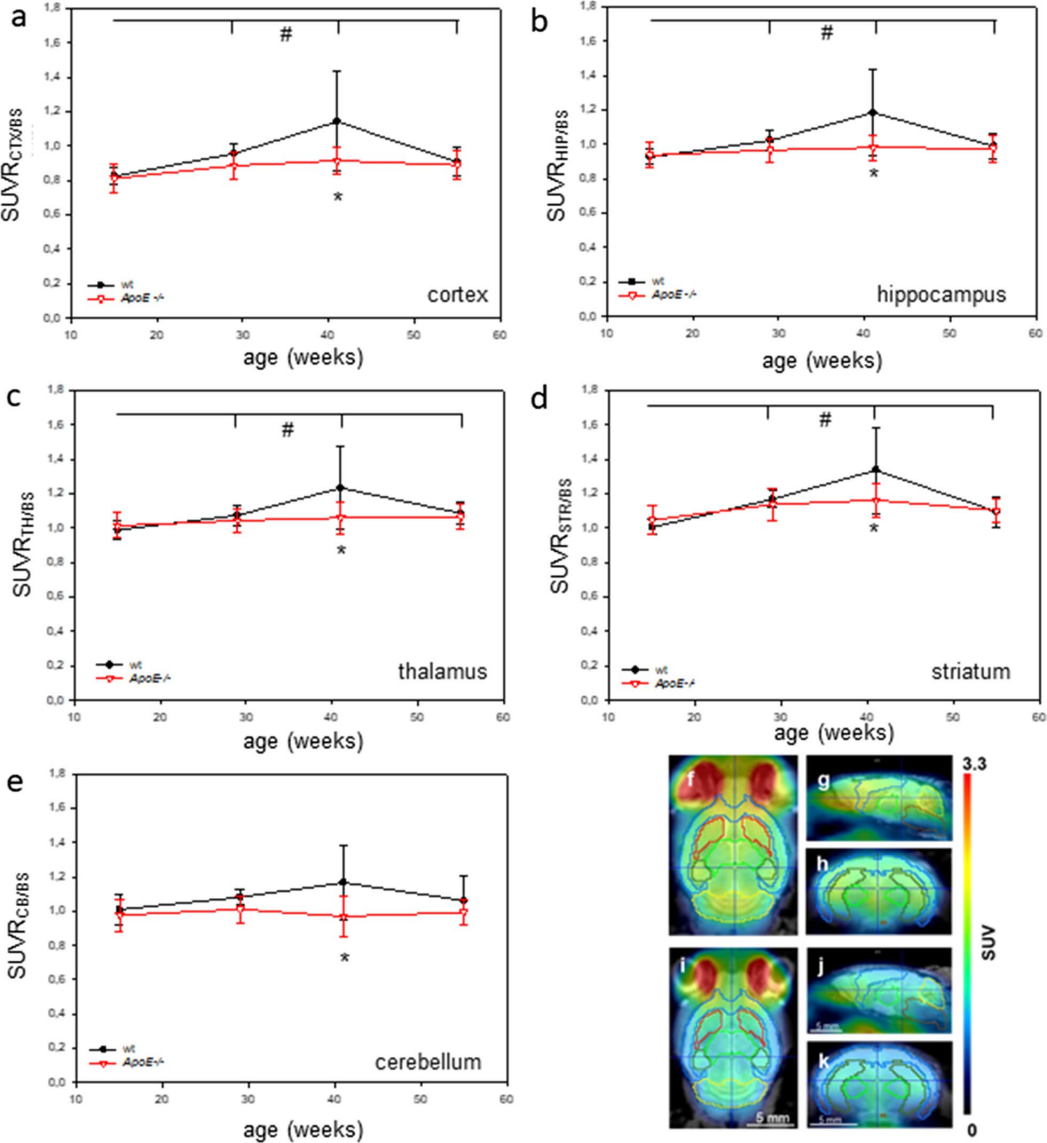
Target-to-brain stem ratio of [^18^F]FDG uptake in the cortex (SUVR_CTX/BS_), hippocampus (SUVR_HIP/BS_), thalamus (SUVR_TH/BS_), striatum (SUVR_ST/BS_), and cerebellum (SUVR_CB/BS_) of wild type (wt; *n =* 8) and Apolipoprotein E-deficient (*ApoE*−/−; *n* = 8) mice (**a**–**e**). Values are given as mean ± SD; ANOVA for repeated measurements followed by Holm-Sidak comparison test: *p < 0.05 versus wt, ^#^*p* < 0.05 versus 15 weeks. Visual comparison of [^18^F]FDG uptake in the brain of wt (**f**–**h**) and *ApoE*−/− mice (**i**–**k**). Transversal (**f**, **i**), sagittal (**g**, **j**), and coronal (**h**, **k**) [^18^F]FDG-PET/CT and MRI with M. Mirrione VOI template overlay and fusion (template: cortex—blue, striatum—red, thalamus—light green, hippocampus—dark green, cerebellum—yellow, brain stem—brown)

Most pre-clinical [^18^F]FDG-PET/CT brain studies used a reference region to evaluate the [^18^F]FDG uptake. SUV data of all examined brain regions are shown in Additional file 2: Fig. 2S. We refrained to show SUVglc (SUVglc = SUV × glc), since the blood sugar concentrations did not differ between the mouse strains as also described by Deleye et al. [32]. Since quantitative analysis of SUV data in the brain stem revealed neither age-dependent nor strain-dependent differences in [^18^F]FDG uptake (Fig. 3), the brain stem was used as reference in the present study. In doing so, analysis of SUVR data revealed a significant time effect in cortex (within-subject type “time-effect”; *p* < 0.001), hippocampus (within-subject factor “time”; *p* = 0.001), thalamus (within-subject factor “time”; *p* = 0.001) and striatum (within-subject factor “time”; *p* < 0.001) (Fig. 4a–d). In addition, Holm-Sidak comparison test revealed a strong age-dependency within wt mice at an age of 41 weeks in cortex, hippocampus, thalamus, and striatum (for detailed statistics see legend of Fig. 4). Furthermore, a significant genotype effect was detected in cortex (between-subject factor “genotype”, *p* = 0.038) and cerebellum (between-subject factor “genotype”, *p* = 0.036). Notably, the mean SUVRs of *ApoE*−/− versus wt mice showed a flatter curve with aging which was significant in cortex (interaction “genotype*time”: *p* = 0.044), hippocampus (interaction “genotype*time”: *p* = 0.024), thalamus (interaction “genotype*time”: *p* = 0.029) and striatum (interaction “genotype*time”: *p* = 0.030). SUVRs of *ApoE*−/− mice were significantly decreased in cortex, hippocampus, thalamus, striatum, and cerebellum at the age of 41 weeks when compared to wt mice (Fig. 4a–e, for detailed statistics see legend of Fig. 4). In line with these data, representative PET images of cerebral uptake of [^18^F]FDG in a 41-week-old wt and *ApoE*−/− mouse visualized reduced [^18^F]FDG uptake in the brain of the *ApoE*−/− mouse (Fig. 4f–h), especially in the cortex, hippocampus, thalamus, striatum, and cerebellum (Fig. 4i–k).

### Characterization of AD pathology

**Fig. 5:**
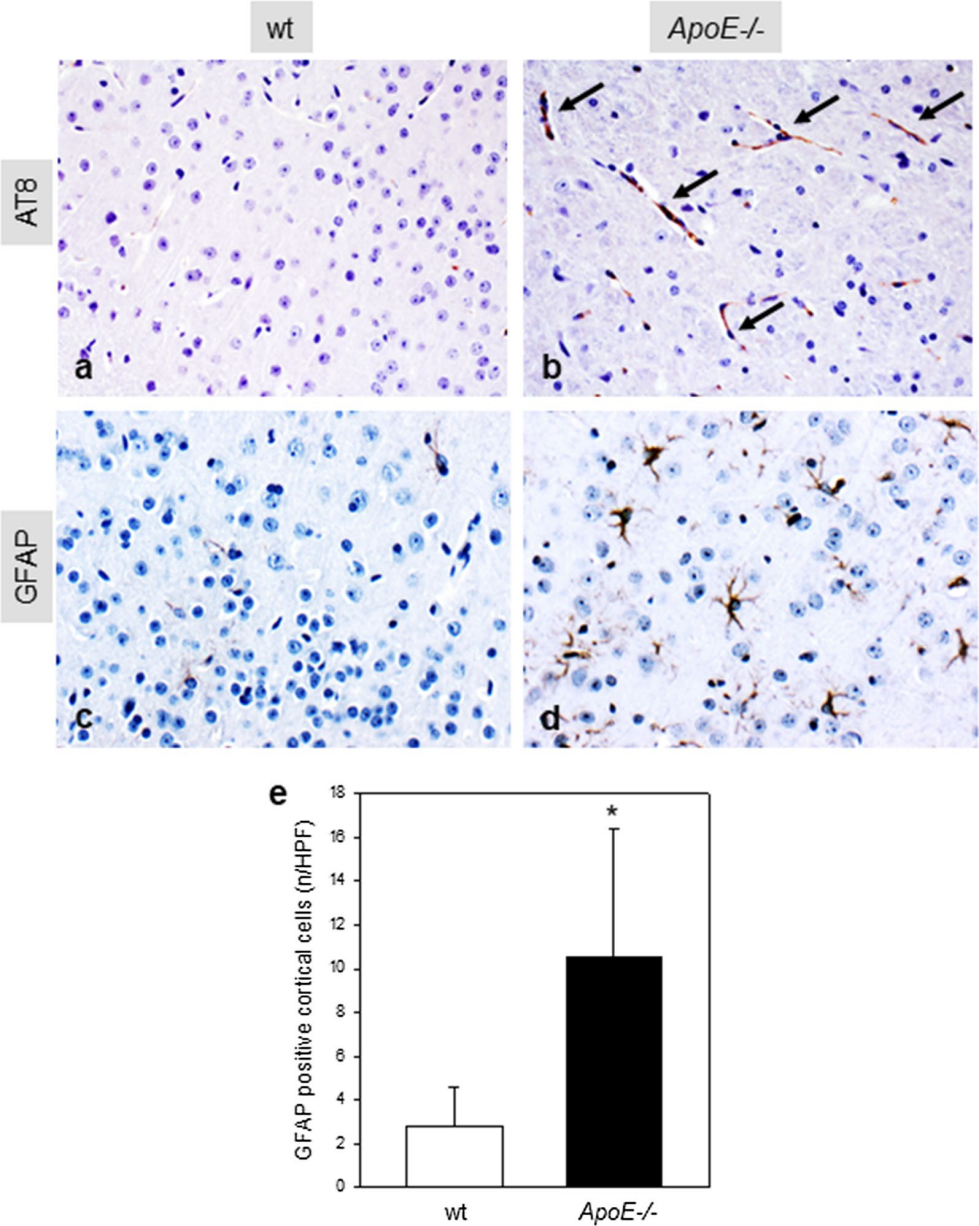
Representative immunohistochemical images (original magnification × 400) of AT8 (**a**, **b**, arrows) and GFAP (**c**, **d**) stained brain sections of each 55 weeks old wild type (wt; **a**, **c**) and Apolipoprotein E-deficient (*ApoE*−/−; **b**, **d**) mouse as well as the quantitative analysis of cortical GFAP-positive cells (**e**, *n* = 8 of each mouse strains) given as number per high power field (HPF). Values are given as means ± SD. Unpaired Student-*t* test followed by Mann–Whitney Rank sum test: *p < 0.05 versus wt

*ApoE*−/−* mice* showed typical signs of tauopathy indicated by increased numbers of AT8 positive cells (Fig. 5b, arrows) when compared to wt mice (Fig. 5a). The analysis of GFAP positive cortical cells demonstrated that *ApoE*−/− mice are characterized by a raised cortical astrogliosis (Fig. 5d), as indicated by an up to sixfold increase of GFAP positive cells when compared to wt mice (*p* = 0.002; Fig. 5e).

## Discussion

AD is characterized by an alteration of the metabolic rate of the cerebral glucose metabolism [33, 34] possibly related to impairment of synaptic activity and followed by late neuronal loss. Interestingly, in cognitively unimpaired ApoE4 carriers the brain glucose hypometabolism which is characteristic for AD precedes the onset of cognitive decline [14, 17]. Consequently, [^18^F]FDG imaging appears as an attractive translational approach to characterize ApoE deficiency related failure of synaptic activity as an early biomarker in an *ApoE*−/− transgenic model.

It is a common approach in pre-clinical [^18^F]FDG-PET/CT studies investigating AD pathologies to use absolute SUVs [18, 35] or SUVglc [32, 36]. However, normalization to an appropriate reference region in [^18^F]FDG-PET imaging is a useful approach to enhance diagnostic performance in neurodegenerative diseases [32, 37]. The cerebellum is in fact widely utilized in [^18^F]FDG-PET research of neurodegenerative dementia [38] because this region is usually not affected by the AD pathology. Accordingly, Poisnel et al. [28] normalized [^18^F]FDG uptake in APPswe/PS1 mice also to cerebellum. Due to the fact that in the current study the brain stem showed neither age-dependent nor strain-dependent differences in [^18^F]FDG uptake this brain region seems to be an ideal reference for normalization in the *ApoE*−/− mouse model. In doing so, we found significant overall effects comparing regional metabolism between *ApoE*−/− and wt mice in cortex, hippocampus, striatum, and thalamus. Post hoc analysis revealed reductions of regional metabolism in all observed brain regions at an age of 41 weeks in *ApoE*−/− versus wt mice. Since peripheral glycemic levels might impact on the degree of brain FDG uptake [39] it is necessary to exclude this potential confounding factor. Though we used non-fasted mice, blood glucose contentrations did not differ between the mouse strains (e.g. 41 weeks old mice wt: 8.3 ± 2.7; *ApoE*−/−: 8.0 ± 2.8). Thus, reduced FDG uptake in *ApoE*−/− mice can certainly be interpreted as impaired glucose metabolism.

The molecular mechanism of metabolic changes in glucose levels in the brain is not fully understood, but is likely related to the malfunction of neuronal glucose transporters or glycolytic enzymes. Furthermore, the metabolic changes could be linked to dysregulation of systemic glucose metabolism in case of impaired cholesterol transport. Comparably, it has been demonstrated that [^3^H]-_D_-glucose transport is reduced up to 29% in hApoE4-TR mice [40]. Accordlingly, these mice showed decreased SUV uptakes in hippocampus and cortex when compared to hApoE2 TR mice [18], which was also shown for the first time in the present study for the *ApoE*−/− mice. In addition, Wu et al. [41] reported that hApoE4-TR mice revealed reduced expression of glucose transporter-3 (GLUT-3). Therefore, it could be speculated that the glucose transporters in *ApoE*−/− mice are also reduced, although a verification is still missing. The protein expression of GLUT3 has been shown to be decreased in parallel with reduced cerebral glucose metabolism in AD-vulnerable brain regions [42]. Therefore, lower GLUT3 expression could lead to insufficient energy supply and perturbation of neuronal function in the brain of ApoE4 carriers. In this context, it is described that hypometabolism of glucose per se is associated with cholesterol-related AD progression [16], which may be linked to ApoE deficiency-induced hypercholesterolemia [43] and impaired glucose metabolism. This is supported by the fact that 27-hydroxycholesterol impairs neuronal glucose uptake via a dysregulation of IRAP/GLUT4 system [44]. In addition, it can also be argued that an altered cholesterol transport per se leads to reduced synaptogenesis due to the absence of ApoE, since cholesterol is required for the repair of synaptic connectivity [5, 6, 45]. In support of this, Zerbi et al. [46] reported a reduction of PSD-95 positive neurons and a strong decline of functional connectivity in *ApoE*−/− mice. Furthermore, *ApoE*−/− mice are characterized by a significant increase of tau-phosphorylation [13] and by enhanced AT8 signal (current study), contributing to the assumption that hypercholesterolemia causes tau hyperphosphorylation [43]. It is known that tau measured in cerebrospinal fluid (CSF) strongly correlates with the [^18^F]FDG signal [47]. Consistently, tauopathy as demonstrated in *ApoE*−/− mice may be contributing to alterations of [^18^F]FDG-PET signal in these mice although other factors such as synaptic dysfunction unrelated to tauopathy may play a role as well. In addition, [^18^F]FDG-PET/CT not only demonstrates synaptic activity, but also neuroinflammatory processes [48], which correlate with TSPO-PET/CT [49]. Accordingly, Poisnel et al. [38] detected an increased [^18^F]FDG uptake in APPswe/PS1 mice, another AD mouse model, characterized by amyloidosis and pronounced neuroinflammation [33, 49, 50]. Moreover, a significant increase of the number of GFAP positive cells was found in the cortex of *ApoE*−/− mice, which reflects enhanced astrogliosis [51] as an indication of present neuroinflammation [52, 53]. It can therefore be assumed that the [^18^F]FDG signal in the *ApoE*−/− mice could be superimposed by neuroinflammatory processes and would even be lower if not masked by inflammation-induced [^18^F]FDG uptake increase.

^1^H-MRS is an additional in vivo technique to characterize metabolic changes in AD brains. A typical metabolite is NAA which has been shown to be decreased in an AD mouse model (APP/PS1 mice) by the group of Chen [24, 54–57] and our group [29]. In addition, the NAA to creatine (Cr) ratio was significantly decreased in these mice [24, 29]. However, the present study could neither show age-dependent nor strain-dependent changes in the NAA/Cr ratio implicating that this biomarker is not meaningful for the characterization of metabolic changes in *ApoE*−/− mice.

Beside transgenic-related alterations of glucose metabolism, even wt mice showed age-dependent changes in glucose metabolism. This was indicated by an initial increase of the [^18^F]FDG signal peaking at 41 weeks and a subsequent decline at an older age, reaching values as found in 55 weeks old *ApoE*−/− mice. This might be due to the physiological aging process as found analogous to human studies [58]. However, Brendel et al. [59] reported that in contrast to findings in the human brain, [^18^F]FDG-PET shows cerebral hypermetabolism of aged wt mice relative to younger animals, supposedly due to microglial activation. Nevertheless, this aspect suggests that the brain glucose metabolism shows age-related changes, independent of the genetic status as described by Ding et al. [60], showing that non-transgenic mice exhibit a significant decrease of [^18^F]FDG uptake already at an age of 36 weeks. This finding is consistent with several human studies demonstrating age-related changes in brain glucose metabolism in healthy adults [61–63].

It is interesting to emphasize, that 55 weeks old *ApoE*−/− mice are characterized by tauopathy with a concomitant increase of astrogliosis, while cerebral hypometabolism was most pronounced at the age of 41 weeks. Based on this, it can be carefully concluded that the [^18^F]FDG signal might be an early biomarker to mirror tauopathy as one hallmark of AD. In line with this, human studies reported that [^18^F]FDG signal is used as an early biomarker representing cerebral metabolic changes before clinical symptoms occur [64].

## Conclusion

In summary, this longitudinal in vivo study shows for the first time that *ApoE*−/− mice depict cerebral hypometabolism without neurochemical alterations. To increase diagnostic sensitivity, a follow-up study with tracers targeting neuroinflammation and tauopathy is recommended.

## Supplementary information

Additional file 1: Fig. 1S Blood glucose concentrations of wild type (wt; *n =* 8) and Apolipoprotein E-deficient (*ApoE–/–*; *n =* 8) mice were measured directly before (A) and after (B) [^18^F]FDG-PET/CT scans. Values are given as mean ± SD

Additional file 2: Fig. 2S Quantification of [^18^F]FDG uptake in the cortex, hippocampus, thalamus, striatum, cerebellum, and brain stem as absolute SUVs of wild type (wt; *n =* 8) and Apolipoprotein E-deficient (*ApoE−/−*; *n* = 8) mice at the age of 15, 29, 41, and 55 weeks. Values are given as mean ± SD; ANOVA for repeated measurements followed by Holm-Sidak comparison test: *p < 0.05 versus 15 weeks

## Data Availability

The datasets used and/or analysed during the current study are available from the corresponding author on reasonable request.

## Abbreviations

AD: Alzheimer's disease
ApoE4: Apolipoprotein E4
[^18^F]FDG-PET/CT: 18-Fluoro-2-deoxyglucose
^1^H-MRS: Magnetic resonance spectroscopy
NAA: *N*-Acetylaspartate
VOI: Brain volume-of-interest
STEAM: Stimulated Echo Acquisition Method
SUVs: Standardized uptake values
ANOVA: Analysis of variance
hApoE-TR: Human APOE 4 isoform
GLUT-3: Glucose transporter-3
HPF: High power field
