# Supplementary figures and images for: Longitudinal [18F]FDG-PET/CT analysis of the glucose metabolism in ApoE-deficient mice
Source: EJNMMI Res. 2020 Oct 7;10:119. doi: 10.1186/s13550-020-00711-4 (PMC7541807; doi:10.1186/s13550-020-00711-4)

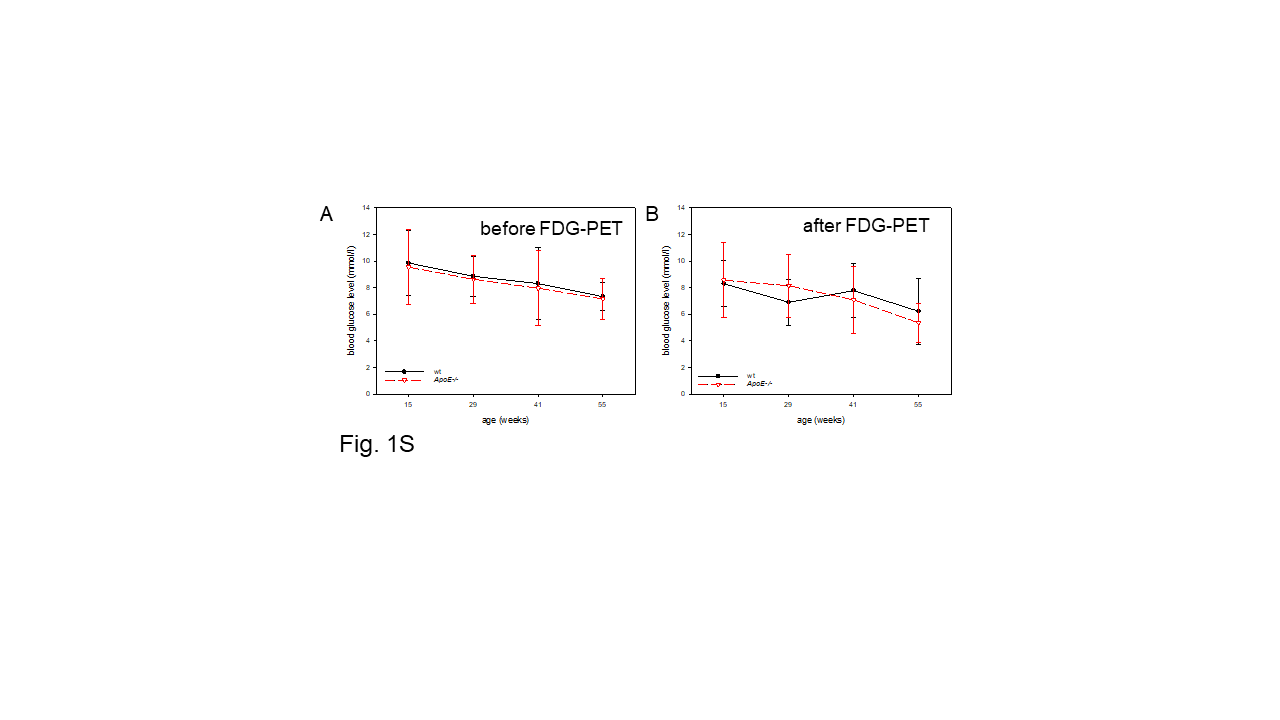

Supplement: Supplementary file 1 — Blood glucose concentrations of wild type (wt; n = 8) and Apolipoprotein E-deficient (ApoE–/–; n = 8) mice were measured directly before (A) and after (B) [18F]FDG-PET/CT scans. Values are given as mean ± SD [file 13550_2020_711_MOESM1_ESM.tif]

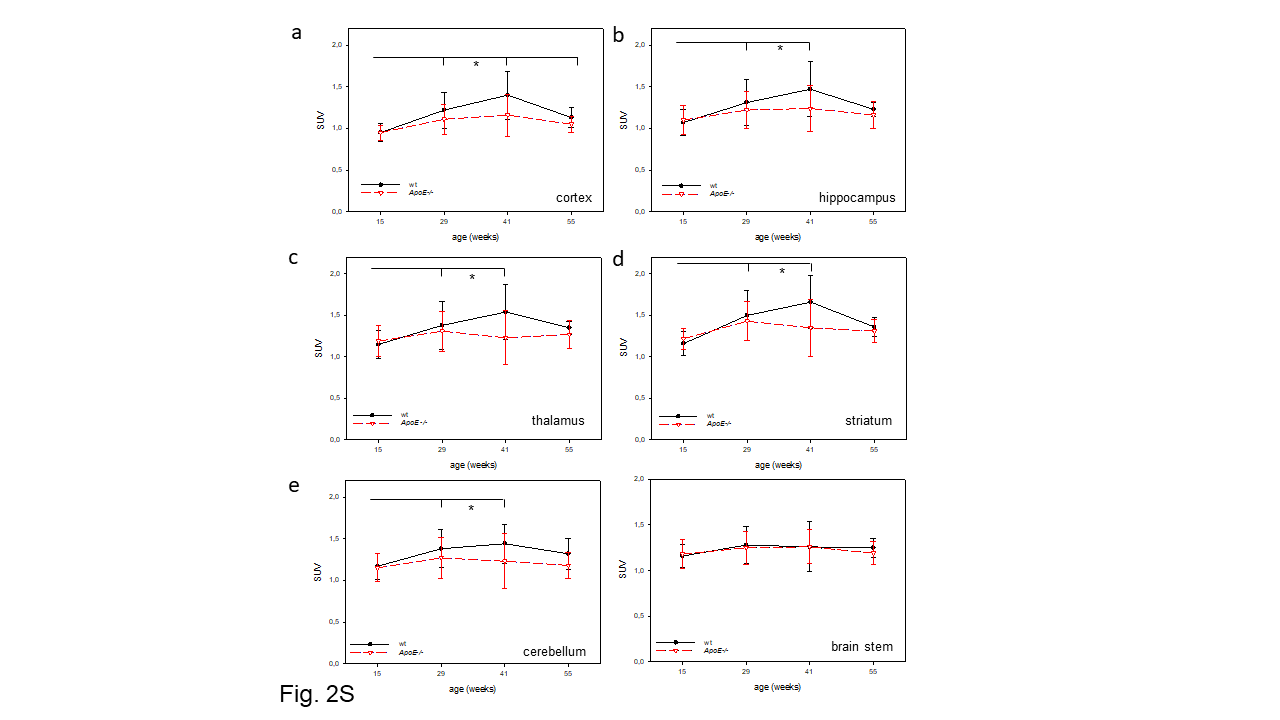

Supplement: Supplementary file 2 — Quantification of [18F]FDG uptake in the cortex, hippocampus, thalamus, striatum, cerebellum, and brain stem as absolute SUVs of wild type (wt; n = 8) and Apolipoprotein E-deficient (ApoE−/−; n = 8) mice at the age of 15, 29, 41, and 55 weeks. Values are given as mean ± SD; ANOVA for repeated measurements followed by Holm-Sidak comparison test: *p < 0.05 versus 15 weeks [file 13550_2020_711_MOESM2_ESM.tif]
